# 

**Published:** 1890-12

**Authors:** 


### Bristol Medico-Chirurgical Journal.

### DECEMBER, 1890.

### CONTENTS.

ORIGINAL PAPERS: *Page*

Multiple Cancellous Exostoses. By EWEN J. MACLEAN, M.B. & C.M. (Edin.) ... ... ... ... ... ... ... ... ... ... ... ... ... ... 217

On Some Varieties of Paraplegia with Lateral Sclerosis, with Cases. By J. MICHELL CLARKE, M.A., M.B. Camb., M.R.C.P. Lond. ... 226

CLINICAL RECORDS:

Some Cases of Peripheral Neuritis. By D. R. PATERSON, M.D. (Edin.), M.R.C.P. (Lond.), Cardiff ... ... ... ... ... ... ... ... ... 244

HEALTH-RESORTS IN THE WEST OF ENGLAND AND SOUTH WALES ... ... 257

REVIEWS OF BOOKS ... ... ... ... ... ... ... ... ... ... ... ... ... ... 268

NOTES ON PREPARATIONS FOR THE SICK ... ... ... ... ... ... ... ... 275

PERISCOPE ... ... ... ... ... ... ... ... ... ... ... ... ... ... ... 278

SCRAPS ... ... ... ... ... ... ... ... ... ... ... ... ... ... ... ... 287

